# Effect of Fatty Acid Composition in Polysorbate 80 on the Stability of Therapeutic Protein Formulations

**DOI:** 10.1007/s11095-021-03125-6

**Published:** 2021-11-29

**Authors:** Melissa A. Pegues, Karol Szczepanek, Faruk Sheikh, Seth G. Thacker, Baikuntha Aryal, Mohamed K Ghorab, Steven Wolfgang, Raymond P. Donnelly, Daniela Verthelyi, V. Ashutosh Rao

**Affiliations:** 1 Laboratory of Applied Biochemistry, Division of Biotechnology Research and Review III, Office of Biotechnology Products, Office of Pharmaceutical Quality, Center for Drug Evaluation and Research, Food and Drug Administrations, Silver Spring, MD 20993 USA; 2 Division of Biotechnology Research and Review II, Office of Biotechnology Products, Office of Pharmaceutical Quality, Center for Drug Evaluation and Research, Food and Drug Administrations, Silver Spring, MD 20993 USA; 3 Laboratory of Immunology, Division of Biotechnology Research and Review III, Office of Biotechnology Products, Office of Pharmaceutical Quality, Center for Drug Evaluation and Research, Food and Drug Administrations, Silver Spring, MD 20993 USA; 4 Policy Development and Evaluation Branch 1, Division of Regulations, Guidance and Standards, Office of Policy for Pharmaceutical Quality, Office of Pharmaceutical Quality, Center for Drug Evaluation and Research, Food and Drug Administrations, Silver Spring, MD 20993 USA; 5 Cosmetics Regulatory Activities Branch, Cosmetics Division, Office of Cosmetics and Colors, Center for Food Safety and Applied Nutrition, College Park, MD 20740 USA

**Keywords:** Polysorbate, stability, immunogenicity, fatty acid, esterase

## Abstract

**Purpose:**

Polysorbate excipients are commonly used as surfactants to stabilize therapeutic proteins in formulations. Degradation of polysorbates could lead to particle formation and instability of the drug formulation. We investigated how the fatty acid composition of polysorbate 80 impacts the degradation profile, particle formation, and product stability under stress conditions.

**Methods:**

Two polysorbate 80-containing therapeutic protein formulations were reformulated with either Polysorbate 80 NF synthesized from a fatty acid mixture that contains mainly oleic acid (≥58%) or a version of polysorbate 80 synthesized with high oleic acid (>98%). Stress conditions, including high temperature and esterase spiking, were applied and changes to both the polysorbate and the therapeutic protein product were investigated for stability, purity, innate immune response and biological activity.

**Results:**

The addition of esterase and storage at 37°C led to significant hydrolysis of the polysorbate and increases in sub-visible particle formation for both polysorbates tested. The fatty acid composition of polysorbate 80 did not directly alter the stability profile of either therapeutic protein as measured by size exclusion chromatography, or significantly impact innate immune response or biological activity. However, formulations with Polysorbate 80 NF showed greater propensity for sub-visible particle formation under stress conditions.

**Conclusions:**

These results suggest that composition of fatty acids in polysorbate 80 may be a promoter for sub-visible particulate formation under the stress conditions tested but may not impact protein aggregation or biological activity.

## Introduction

Polysorbates are a family of excipients that function as surfactants. These non-ionic amphipathic surfactants are composed of polyethoxylated sorbitan fatty acid monoesters in which the fatty acid composition is heterogeneous. Different commercial grades of Polysorbate NF are defined in the United States Pharmacopeia National Formulary (USP-NF) monographs and each grade is differentiated based on the most prevalent fatty acid in the respective fatty acid composition. Polysorbate NF grades are typified by fatty acid chain lengths ranging between 12 and 18 carbon atoms and include Polysorbate 80 NF, in which the most prevalent fatty acid is monounsaturated. Polysorbates are commonly used in therapeutic protein formulations where they act as stabilizers to prevent protein adsorption at surface interfaces. They may also prevent protein aggregation through specific interaction with the aggregation-prone hydrophobic regions of a protein molecule, thus preventing self-association (1–3). Polysorbate 20 NF and Polysorbate 80 NF are the most frequently used surfactants in therapeutic protein formulations to help prevent aggregation or degradation of the protein and a polysorbate concentration of 0.001% to 0.01% typically provides the desired effect (4, 5). Lauric acid (a saturated, C-12 fatty acid) and oleic acid (a monounsaturated, C-18:1 fatty acid) are the main fatty acid constituents in Polysorbate 20 NF and Polysorbate 80 NF, respectively (4, 6). Each Polysorbate NF monograph specifies a range for each of the various fatty acid constituents to accommodate variation in naturally occurring oils serving as the source of fatty acids used to synthesize this excipient. This tendency for natural variation results in a heterogeneous fatty acid composition and the potential for significant lot-to-lot variability of the fatty acid composition in polysorbate excipients found in therapeutic protein formulations. Improved understanding about how the variability of the fatty acid content in polysorbate excipients impact the stability of therapeutic protein formulations will enable manufacturers to optimize quality and safety of protein drug formulations.

Polysorbate degradation could have a negative impact on the quality and safety profiles of a drug product. Degradation of polysorbate could impair its ability to function as a stabilizer of therapeutic proteins (7, 8). Degradation or aggregation of the therapeutic protein could in turn lead to unexpected immunogenicity or loss of therapeutic efficacy or both (9, 10). Polysorbate is known to be susceptible to multiple degradation pathways, including autoxidation (4, 11) and hydrolytic cleavage (12). The latter route of degradation to form free fatty acid (FFA) may lead to formation of visible or subvisible particles in formulated injectable therapeutic proteins. One potential source of fatty acids in the observed particles are the residual FFA that may be present in the polysorbate excipient before it is used to manufacture a drug product (13). FFA can also arise from reaction of polysorbate with therapeutic protein manufacturing process-related impurities or components of the formulation such as buffers, solvents, and residual host cell enzymatic proteins (14–17). Previous publications have reported observation of visible and sub-visible particles in some formulations and the apparent decreases in polysorbate concentration over time under conditions where enzymatic host cell proteins were identified as the mediator of hydrolysis of the fatty acid ester (14). Propensity for polysorbate to form fatty acid particles appears to increase, perhaps due to a decrease in solubility, as fatty acid chain-length increases (13). However, the relationship between the fatty acid composition of polysorbate, content of FFA in the drug formulation, propensity for particle formation, and critical protein quality attributes is complex and still not completely understood.

To address this, we investigated whether the purity in the fatty acid composition of polysorbate 80 (i.e., oleic acid content) affects particle formation and therapeutic protein stability in formulations subjected to stress conditions, whereby degradation of the polysorbate and subsequent interactions involving FFA and protein may be accelerated. We compared a commercially available Polysorbate 80 NF excipient having a heterogeneous fatty acid composition in which the oleic acid content was >58% (NF specification: 58% oleic acid, minimum) and a non-commercial analog in which oleic acid comprises >98% of the fatty acid composition. Two different therapeutic protein formulations, recombinant human granulocyte colony stimulating factor (rhG-CSF) and rituximab (an anti-CD20 monoclonal antibody), were formulated with the two types of polysorbate. The formulation for rhG-CSF contained 0.004% (w/v) Polysorbate 80 whereas the Rituximab formulation contained a Polysorbate 80 concentration of 0.07% (w/v). The formulations were prepared with and without the therapeutic proteins, subjected to stress conditions (heat and addition of esterase) and monitored for chemical stability, particle formation, biological activity, and immunogenic potential.

## Materials and Methods

### Chemicals and Reagents

Polysorbate 80 NF was purchased from Spectrum Biochemical. Super refined polysorbate 80 with C18:1 fatty acid content ≥98% designated as “high oleic acid PS80 (PS80 HOA)” throughout this manuscript, was kindly provided by Croda Inc. rhG-CSF was purchased as filgrastim reference standard from USP and rituximab from Genentech, Inc. Porcine liver esterase was purchased from Sigma. Mouse phospholipase B-like 2 (PLBD2) was purchased from Biorbyt. All chemicals were purchased from Sigma unless otherwise specified.

### Sample Preparation

For measurement of FFA and particulates, Rituximab and rhG-CSG formulation buffers were prepared without adding Rituximab or rhG-CSG in the formulation buffers. To assess the impact of enzymatic hydrolysis of PS80 on stability, biological activity and immunogenicity of therapeutic proteins, each therapeutic protein was sufficiently diluted in formulation buffer without polysorbate 80 to maintain polysorbate 80 concentration from the original formulation of each drug below its CMC value of 0.0017%. At final round of dialysis, each drug was diluted and dialyzed against formulation buffer prepared with either Polysorbate 80 NF or polysorbate 80 HOA at the indicated concentration. rhG-CSG (0.6 mg/ml) was prepared in 10 mM sodium acetate, 274.5 mM sorbitol, with 0.004% polysorbate 80 at pH 4.0. Rituximab (10 mg/ml) was prepared in 25 mM sodium citrate, 154 mM sodium chloride, 0.07% polysorbate 80 at pH 6.5. All formulations were filtered using a 0.2 μm Millex-VV syringe filter unit prior to experimentation. Formulation prepared with and without therapeutic proteins were spiked with 1 U/mL esterase and both esterase-spiked and unspiked (control) samples were stored at 4°C, 25^o^C or 37°C.

### Measurement of FFA

Total FFA release was measured using the enzymatic colorimetric Non-Esterified Fatty Acid assay (NEFA) kit from Wako Diagnostics according to the manufacturer’s instructions. This method relies on the acylation of coenzyme A (CoA) and oxidation of COA by acyl-CoA oxidase to produce hydrogen peroxide. The added peroxidase (POD) then allows for the oxidative condensation of 3-methyl-N-ethyl-N-(ß- hydroxyethyl)-aniline (MEHA) with 4-aminoantipyrine to form a purple-colored end product which can be measured calorimetrically at 550 nm. The assay has broad linearity range from 0.01–4.00 mEq/L. While it is not possible to calculate exact concentration of FFA release from PS80, we calculated expected maximal, theoretical FFA concentration released (the total FFA level expected when all fatty acid esters undergo hydrolytic cleavage) for each formulation by assuming a fatty acid composition of 100% oleic acid in PS80 and considering release of one FFA per PS80 molecule. This is indicated by a dashed line in each figure.

### Rituximab Activity Via Antibody Dependent Cellular Cytotoxicity (ADCC)

ADCC activity was measured using the Promega ADCC Reporter Bioassay following the manufacturer’s instructions. CD20^+^ RAJI target cells were purchased from Promega and maintained at 37°C in a humidified 5% CO_2_ atmosphere in RPMI-1640 medium (Corning) supplemented with 10% FBS (Gemini Bio-Products), 2 mM L-glutamine, and 1 mM sodium pyruvate; Effector cells were supplemented with 100 μg/mL hygromycin, 250 μg/mL G-418 sulfate, and 0.1 mM MEM non-essential amino acids. Reformulated rituximab samples were diluted to 3 μg/mL in ADCC assay buffer (RPMI-1640 supplemented with 2 mM l-glutamine and 0.5% low-IgG FBS). Serial dilutions gave a range of 1 μg/mL to 15.2 pg/mL final antibody concentrations for each sample. Assays were performed in 96-well plates with an effector to target ratio of 150,000:30,000 cells/well. Luciferase activity was measured after incubation at 37°C for 18 h using Bio-Glo Luciferase Assay System (Promega) on a SpectraMax i3 microplate reader (Molecular Devices). Data were analyzed as fold-induction of luminescence compared to a no-antibody control and the EC_50_ was determined by fitting log_10_([antibody, μg/mL]) versus response using GraphPad Prism. Data are reported as three independent experiments.

### rhG-CSF Activity Via Phosphorylation of STAT3. rhG-CSF

biological activity was measured by induction of STAT3 phosphorylation in 32D cells that were stably transfected with the wild-type, full-length, human G-CSF receptor gene (18). Briefly, each test sample was diluted to 1 ng/mL and applied to 32D-G-CSFR cells for 30 min at 37°C before the cells were harvested, washed, and lysed. The whole cell lysates were then assessed by western blotting for STAT3 and p-STAT3 (Y705). The positive control (reference) sample was derived from 32D-G-CSFR cells treated with recombinant human G-CSF at 1 ng/mL for 30 min at 37°C. The levels of tyrosine-phosphorylated-STAT3 were calculated as a ratio to total STAT3 levels after densitometric quantitation.

### Particle Measurement

Particles (1–150 μm diameter) were measured using microfluidic imaging (MFI) 5200 Flow Microscope (ProteinSimple, San Jose, Ca) equipped with a 100 μm flow cell. Samples were analyzed at 0.15 mL/min flow rate, and particles were reported as number of particles/mL.

### Chromatography

Size exclusion (SEC) and reverse phase (RP) chromatography was performed using a Waters Acquity UPLC System (Milford, MA, USA) equipped with UV and fluorescence detectors. SEC-UPLC separation was performed using an Acquity UPLC Protein BEH SEC 200 Å column (4.6 × 150 mm; 1.7 μm) using mobile phase 50 mM sodium phosphate, 150 mM NaCl, pH 6.8 for Rituximab and 0.1 M phosphoric acid, pH 2.5 for rhG-CSF at a flow rate of 0.3 mL/min. RP-UPLC separation was performed using an Acquity BEH-300 C18 column with a particle size of 1.7 μm (150 × 2.1 mm, 300 Å) purchased from Waters. Proteins were eluted from the column using a gradient consisting of Mobile phase A: 0.1% TFA in water and Mobile phase B: 0.1% TFA in acetonitrile. Rituximab was eluted with a gradient from 33% to 39% mobile phase B over 6 min and rhG-CSF was eluted using a gradient from 52% to 74.5% mobile phase B over 9 min. UV absorption was collected at wavelength 280 nm.

### Innate Immune Activation

Human peripheral blood mononuclear cells (PBMCs) were used to assess immunogenicity of each formulation. PBMCs were treated with each formulation diluted to a final concentration of 10% in DMEM with 10% FBS for 18 h. PBMCs treated with 10 and 100 pg LPS (0.01 and 0.1EU/ml respectively) were included as a positive control. Cells were then collected and expression of IL-1β and CCL2 mRNA was measured by qPCR. PBMCs from 6 different donors were used to test innate immune stimulatory capacity of stressed polysorbate samples.

### Statistical and Correlation Analyses

Data were analyzed by GraphPad Prism. Statistical significance was determined by student’s T test of analysis of variance (ANOVA). A *p* value of <0.05 indicated statistical significance.

## Results

### Esterase Treatment Leads to Polysorbate Degradation and Particle Formation

**Fig. 1:**
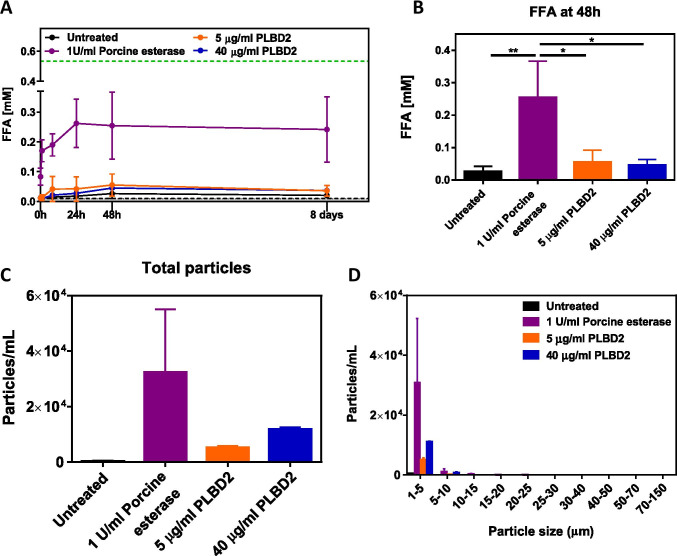
Comparison of the effect of hydrolyzing enzymes. Rituximab formulations were prepared with polysorbate 80 NF and treated with either porcine liver esterase or PLBD2 and stored at 37°C. (A) FFA release over the course of 8 days, (B) maximal levels reached after 48 h of storage (n ≥ 3). (C) Total particles formed after 9 days of storage and (D) particle size distribution for all formulations (n = 2). Data is presented as mean ± SD. *p < 0.05, **p < 0.01

We first evaluated stress conditions that led to significant degradation of polysorbate. Previous reports have identified many host cell proteins with esterase activity that may lead to degradation of polysorbate (14–16). We compared the activity of two of those enzymes, phospholipase B-Like 2 protein (PLBD2) and esterase from porcine liver, to identify stress conditions that would lead to significant and rapid degradation of polysorbate 80 in our chosen formulations. Rituximab formulation was prepared with Polysorbate 80 NF and treated with 1 U/mL porcine liver esterase, 5 μg/mL PLBD2, or 40 μg/mL PLBD2 and stored at 37°C. FFA release and particle formation were monitored and compared to rituximab formulation that was not treated with any hydrolyzing enzymes (Fig. 1). Although PLBD2 led to slight increase in FFA concentration, the increase was not statistically significant from the untreated formulation. The formulation treated with porcine liver esterase showed significant increase in FFA concentration (Fig. 1A, B). This also trended with a corresponding increase in particle formation (Fig. 1C, D). These results highlight the differing propensities of host cell proteins for degradation of polysorbate 80. Because porcine liver esterase treatment showed greater polysorbate 80 degradation over PLBD2, as reflected by higher FFA and particle formation, it was chosen for further use in the enzyme induced stress condition studies conducted.

**Fig. 2:**
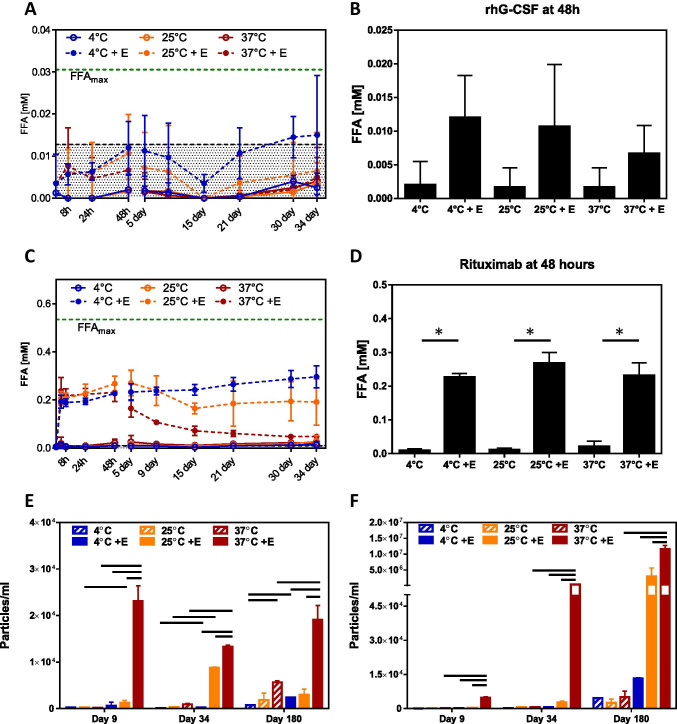
Degradation of polysorbate 80 under accelerated stress conditions. Drug formulations were prepared without therapeutic protein then treated with or without esterase and stored at 4°C, 25°C, or 37°C. The release of FFA over 34 days(A) and maximal FFA release at 48 h (B) was measured for rhG-CSF formulation. The release of FFA over 34 days (C) and maximal FFA release at 48 h (D) was monitored in rituximab formulation during storage at different conditions. Particle formation was monitored for 6 months and total particles (1–150 μm) were measured by MFI for rhG-CSF (E) and rituximab (F) during storage. Data presented as mean ± SD (n = 3–5). Shaded area in fig. 2A represents the limit of detection (LOD) for FFA, Bars indicate *p* < 0.05

To determine the polysorbate 80 degradation profile in formulation, rituximab and rhG-CSF formulations were prepared without the therapeutic protein but with Polysorbate 80 NF, as per the manufacturers’ formulation (i.e., 0.07% w/v and 0.004% w/v for the Rituximab formulation and rhG-CSF formulation, respectively) (18, 19). Both formulations were either treated with 1 U/mL of esterase or untreated and stored at either 4°C, 25°C, or 37°C for up to 6 months. FFA and subvisible particle levels were monitored at multiple timepoints throughout the study for both formulations. The increase in FFAs content observed with the esterase treated rhG-CSF formulation was not statistically significant from the non-treated samples and the total FFA detected for rhG-CSF formulation was below the limit of detection (LOD) even after esterase spiking (Fig. 2A and B) at all storage conditions. On the other hand, rituximab’s formulation showed a significant increase in FFA content at all temperatures when treated with esterase (Fig. 2C and D). The enzyme treated rituximab formulation also showed greater increase in FFAs than the enzyme treated rhG-CSF, which might be due to higher concentration of Polysorbate 80 NF in the rituximab formulation (0.07% w/v) than rhG-CSF (0.004% w/v). No significant increase in FFAs was detected in the samples without esterase treatment over 34 days at all storage temperatures (Fig. 2A and C). It is noteworthy to mention that none of the formulations, even the 1 U/mL esterase treated ones, showed increase in the free fatty acid content that reach the maximal theoretical free fatty acid release (dashed green line in Fig. 2A and C). We used 1 U/mL esterase concentration based on previous publication but did not increase esterase concentration esterase concentration to demonstrate if PS-80 undergoes complete hydrolysis at higher esterase concentration (16). After the initial increase in FFA levels, a subsequent temperature dependent decrease followed, with samples stored at 37°C showing the most rapid decrease.

Particle formation was measured by microflow imaging (MFI) at the indicated time points for all formulations. Increases in total visible and subvisible particles (1–150 μm) were detected only for the esterase treated samples for both rhG-CSF (Fig. 2E) and rituximab (Fig. 2F) formulations and was found to be greatest in samples stored at 37°C.

These results indicated that polysorbate, at the two different concentrations in both rituximab and rh-GSF formulations, is susceptible to hydrolytic cleavage by esterase. Furthermore, the kinetics of FFA levels and particle formation appear to be correlated and temperature dependent.

### Differences in Fatty Acid Composition Lead to Differential Rates of Subvisible Particle Formation

**Fig. 3:**
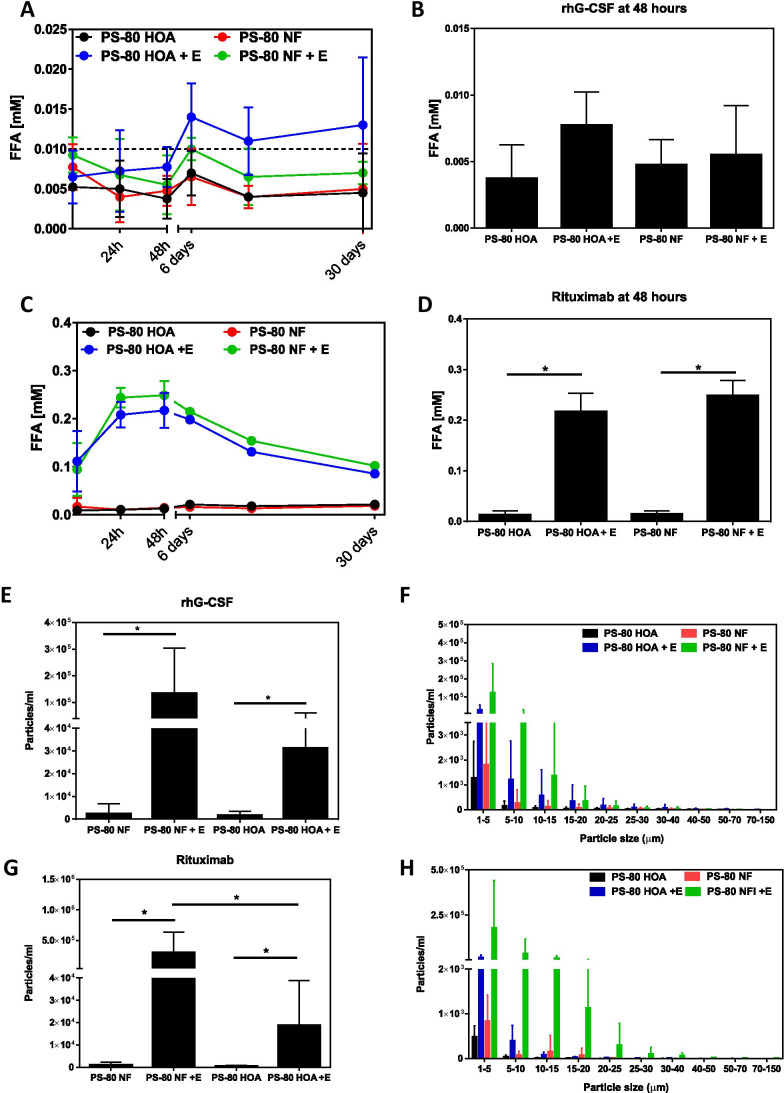
Comparison of polysorbate 80 HOA and Polysorbate 80 NF under stress conditions. Rituximab and rhG-CSF formulations without therapeutic protein were prepared using either Polysorbate 80 NF or polysorbate 80 HOA. Formulations were treated with or without esterase and stored at 37°C. FFA release was monitored over 30 days (A) and maximal FFA release was measured at 48 h (B) for rhG-CSF formulations during storage. FFA release was monitored over 30 days (C) and maximal FFA release was measured at 48 h (D) for rituximab formulation during storage. Total particle formation (E) and distribution of 1–150 μm particle sizes (F) for rhG-CSF formulation at day 30 was measured by MFI. Total particle formation (G) and distribution of 1–150 μm particle sizes (H) for rituximab formulation at day 30 was measured by MFI. Data presented as mean ± SD (n = 4–5). *p < 0.05

We next compared the stability of Polysorbate 80 NF and polysorbate 80 HOA under stress conditions. We measured FFA particle formation at 4°C, room temperature, and at 37°C and observed greater number of particle formation at 37°C. Therefore, to reflect the accelerated stability conditions and for physiological relevance, we focused our study at the accelerated stability condition of 37°C. Based on our previous studies on therapeutic protein stability, we included 37°C as a physiologically relevant temperature with implications on in-use stability during drug administration. Therapeutic protein-free formulations were prepared for rhG-CSF and rituximab using either Polysorbate 80 NF or polysorbate 80 HOA and then subjected to stress conditions. Total FFA release and particle formation was measured after 30 days of storage. The increase in FFAs content observed with the esterase treated rhG-CSF formulations was not statistically significant from the non-treated samples for both Polysorbate 80 NF and polysorbate 80 HOA formulations (Fig. 3A and B). Enzyme treated polysorbate 80 HOA formulation; however, showed more FFAs release than polysorbate 80 NF formulation after 48 h of incubation at 37°C. On the other hand, both Polysorbate 80 NF and polysorbate 80 HOA in rituximab formulations showed a statistically significant increase in FFA content when treated with esterase for up to 30 days (Fig. 3C and D). The maximum enzymatic release of FFAs was observed at 48 h and then FFA level was decreased. While we cannot rule out inactivation of enzyme at 37°C over time, decrease in FFA in our experiment cannot be explained due to inactivation of enzyme because maximum FFA is comparable at all storage conditions and FFA concentration is unchanged over a month at 4°C. The decrease in FFA is expected to be due to due to oxidative degradation of fatty acids (6, 20). Particle formation was also found to increase in the esterase treated rhG-CSF and rituximab formulations formulated with both polysorbate 80 NF and polysorbate 80 HOA (Figs. 3E- 3H). Although the levels of FFA at various time points are similar for both Polysorbate 80 NF and polysorbate 80 HOA in each drug formulation, rituximab formulations containing Polysorbate 80 NF showed significantly higher levels of sub-visible particles than formulations containing polysorbate 80 HOA when treated with esterase (Figs. 3G).

### Fatty Acid Composition of Polysorbate 80 Does Not Affect rhG-CSF Stability

**Fig. 4:**
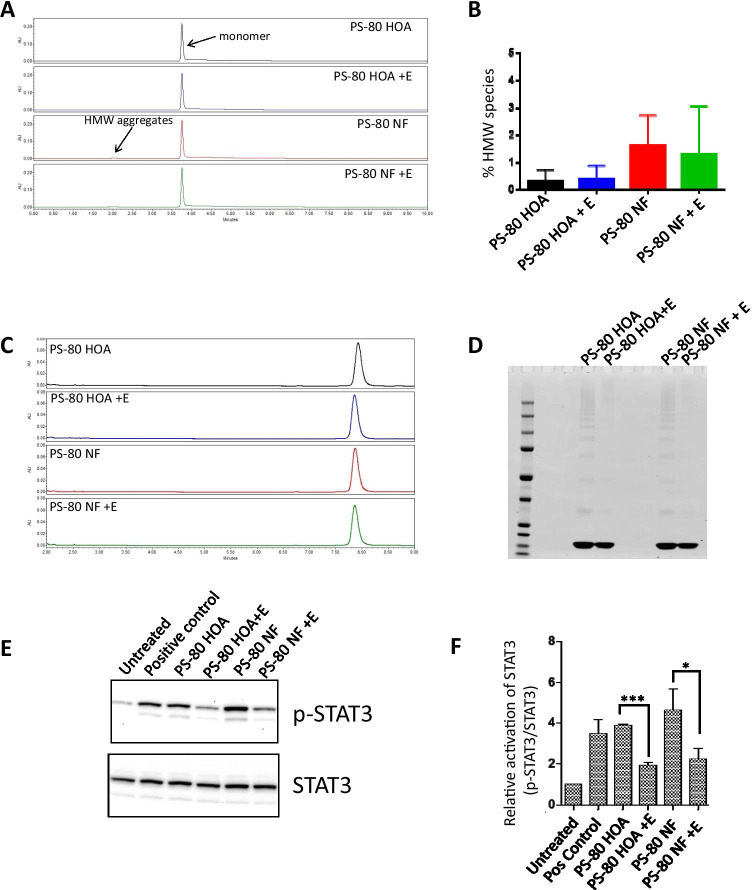
Stability of rhG-CSF therapeutic protein under stress conditions. rhG-CSF formulations containing therapeutic protein were prepared using either Polysorbate 80 NF or polysorbate 80 HOA and stored at 37°C. Monomer integrity was measured by SEC-UPC (A) and a representative size exclusion chromatogram of detection at UV 280 nm at day 30 post-reformulation is shown. (B) The percent high molecular weight species detected by SEC-UPLC was calculated. (C) A representative reverse phase chromatogram detected at UV 280 nm at day 30 post-reformulation. (D) Protein integrity was also measured by SDS-PAGE and a representative Coomassie stained non-reducing gel of rhG-CSF samples at day 30 post reformulation is shown. (E) Representative pSTAT3 immunoblot showing activity of therapeutic protein at day 30 post reformulation and (F) bar graph showing average biological activity as calculated by p-STAT3/STAT3. Data is presented as mean ± SD. *p < 0.05, ***p < 0.001

Protein stability was then assessed in formulations containing the active pharmaceutical ingredient (API) protein prepared with either Polysorbate 80 NF or polysorbate 80 HOA and stored at 37°C with and without 1 U/mL esterase. rhG-CSF protein from formulations with API included in the samples were analyzed by SEC-UPLC (Fig. 4A) and by non-reducing SDS-PAGE (Fig. 4D) to assess protein integrity and aggregation at day 30 after reformulation and storage at 37°C. Although some high molecular weight species were detected after exposure to stress conditions, no significant difference was detectable between formulations prepared with the two versions of polysorbate 80 (Fig. 4A, B). To further assess any charge-based changes, rhG-CSF was analyzed by RP-UPLC, but no significant differences were found (Fig. 4C). Biological activity of rhG-CSF in the formulation after exposure to stress conditions was determined by its ability to induce STAT3 phosphorylation (Fig. 4E). The positive control (reference) sample used in lane-2 was derived from 32D-G-CSFR cells treated with recombinant human G-CSF at 1 ng/mL for 30 min at 37°C. As expected, phosphorylation of STAT3 was significantly increased after treatment with rhG-CSF (positive control) and similar activity levels were observed for two formulations tested. We did not observe significant differences between the two formulations in the absence of esterase; however, we did observe significant decrease in the levels of phosphorylated-STAT3 after treatment with esterase (E) in both the PS80 and PS80 HOA samples. To examine if the stressed polysorbates could potentially increase immunogenicity risk by inducing an innate immune or proinflammatory response, PBMCs were exposed to the stressed formulations. While the difference between the stressed and unstressed samples’ ability to activate human PBMC cells was not statistically significant, some samples showed a modest increase in IL-1β and CCL2 mRNA induction indicates that the stressed samples could pose a modest risk of inducing activation of PBMCs in some subjects (Fig. 6A and B) for both the stressed Polysorbate 80 NF and polysorbate 80 HOA formulations.

### Rituximab Stability and Activity Are Not Affected by Storage in Formulation with either Polysorbate 80 NF or Polysorbate 80 HOA

**Fig. 5:**
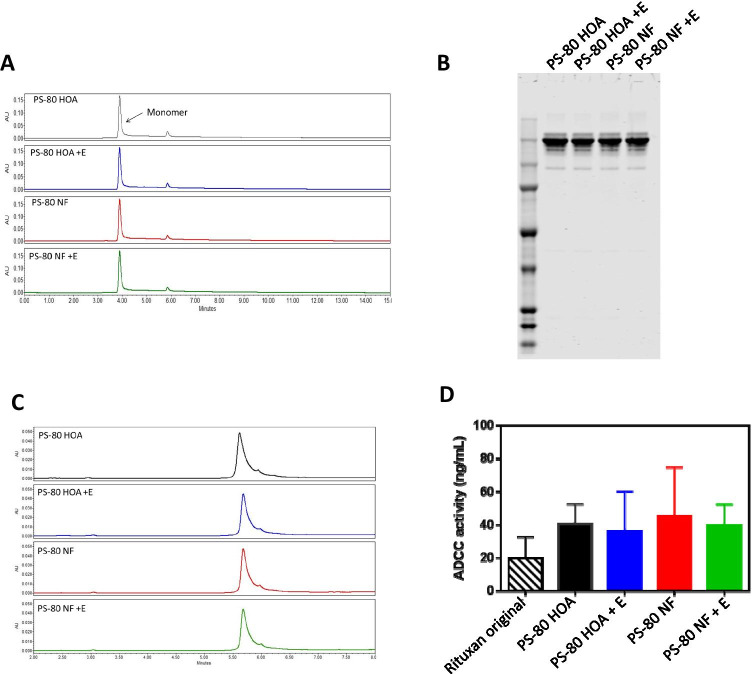
Stability of rituximab therapeutic protein under stress conditions. Rituximab formulations containing therapeutic protein were prepared using either Polysorbate 80 NF or polysorbate 80 HOA and stored at 37°C. for 30 days. (A) Representative size exclusion chromatogram with detection at UV 280 nm at day 30 post-reformulation. (B) Representative Coomassie stained non-reducing SDS-PAGE gel at day 30 post-reformulation. (C) Representative reverse phase chromatogram with detection at UV 280 nm at day 30 post-reformulation. (D) ADCC activity at day 30 post-reformulation shown as mean + SD (n = 3–4)

**Fig. 6:**
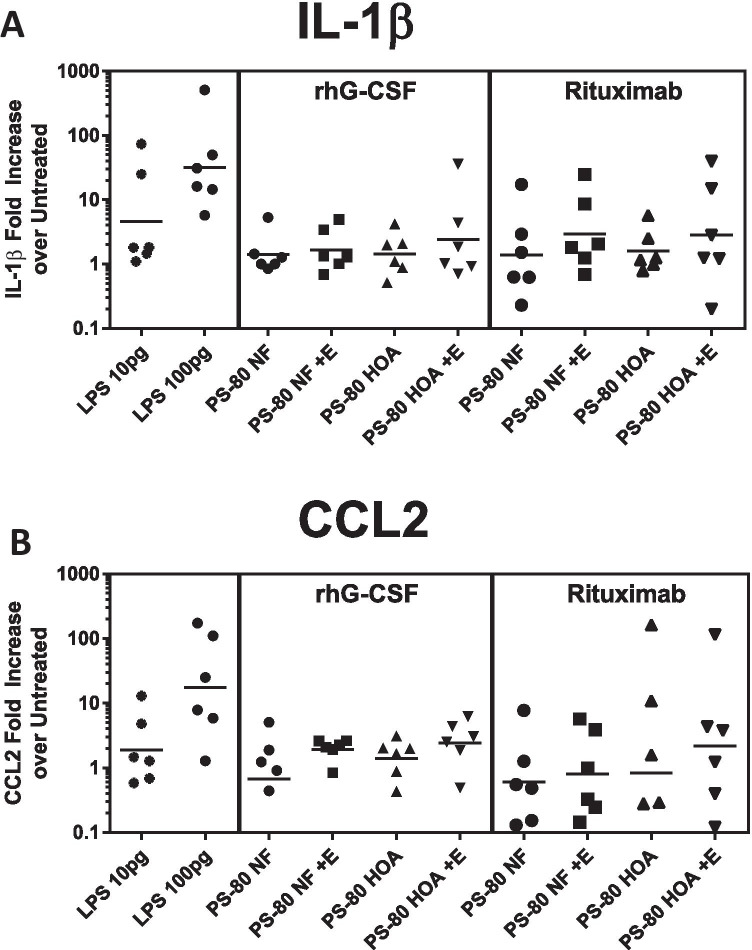
Innate immune activation of PBMC by polysorbate 80 containing formulations. Immunogenicity was assessed by stimulating PBMCs from 6 healthy donors with 10% formulation (v/v) of rhG-CSF and rituximab formulations. Activation was assessed by monitoring induction of IL-1β (A) and CCL2 (B). Graphs show fold change over untreated PBMCs

Rituximab biological activity and stability were also assessed at day 30 after reformulation with either Polysorbate 80 NF or polysorbate 80 HOA in presence or absence of 1 U/mL esterase. No significant differences in size or charge variants were detected after exposure to stress conditions between any formulation when assessed by SEC-UPLC (Fig. 5A), SDS-PAGE (Fig. 5C), or by RP-UPLC (Fig. 5B). ADCC was measured at day 60 to assess biological activity of rituximab after reformulation and compared to the original formula (Fig. 5D). No significant difference in activity was observed between formulations, suggesting that all samples retained activity and that the activity was not affected by polysorbate 80 fatty acid composition or after hydrolytic cleavage of fatty acids. The measurement of innate immune responses in PBMC showed a no increase in CCL2 and IL1β compared to unstressed samples (Fig. 6A and B). Similar to the observation with rhG-CSF above, while the overall magnitude of the response to the rituximab formulation and the difference relative to Polysorbate 80 NF formulated samples were small and not statistically significant, however an increased response in some PBMC samples suggests that a higher response could occur in some subjects.

## Discussion

Compendial specifications for pharmaceutical grades of polysorbate excipients place limited restriction on the purity of the fatty acid and thus allow for lot-to-lot variation in this class of excipients that could adversely affect the quality of therapeutic protein formulations (4). In this study, we evaluated the relationship between two polysorbate 80 fatty acid excipients in which the respective fatty acid compositions have pronounced variation in oleic acid content and therapeutic protein formulation stability, potency, and innate immune response. We tested whether these differences in fatty acid composition affect degradation of the polysorbate and the stability of therapeutic proteins. Polysorbates were susceptible to degradation under stress conditions that included storage at 37°C and spiking with esterase.

Numerous factors, including impurities from raw materials, storage temperature, other excipients, and exposure to light (17, 21), can affect the quality and performance of polysorbate excipients in therapeutic protein formulations. Oxidation and hydrolysis are the two main degradation pathways of polysorbate. Several studies have identified a variety of host cell enzymes causing polysorbate to undergo rapid degradation involving hydrolysis of the fatty acid chain leading to particle formation in polysorbate-containing therapeutic protein formulations (14–16, 22, 23). Furthermore, many of these enzymes are found in expression systems used for therapeutic protein production, including *E. coli*., CHO cells, and yeast, and some research groups are attempting to generate cell lines without these enzymes that could jeopardize the quality of the final product (24–26). For these reasons, we chose to evaluate how these therapeutic protein manufacturing process-related impurities could affect the degradation profiles of two different samples of polysorbate 80 having fatty acid compositions differing most notably in respective content of oleic acid. Many of these enzymes that have been shown to lead to degradation of polysorbates have been shown to do so at varying rates and lead to varying degrees of hydrolysis. Dixit et al. identified PLBD2 as a potential host cell protein that could be responsible for degradation of polysorbate 80 in a mAb-containing formulation. To identify stress conditions that would lead to rapid and significant levels of polysorbate degradation, we compared the ability of PLBD2 and porcine liver esterase to hydrolyze polysorbate. Although PLBD2 is a CHO host cell protein with relevance to bioprocessing and was able to hydrolyze polysorbate 80 in this study, we found that esterase from porcine liver results in a more rapid and higher level of FFA release (Fig. 1). Therefore, the latter enzyme was chosen for the rest of this study. Using this approach, we found that when subjected to stress conditions that included storage at elevated temperatures and exposure to esterase, both types of polysorbate 80 included in this study showed significant degradation as demonstrated by release of total FFA levels at various timepoints throughout storage at 4°C and accelerated storage conditions. Interestingly, we identified differential kinetics for FFA levels that were dependent upon incubation temperature. All formulations treated with esterase showed a substantial increase in FFA levels at early timepoints. However, FFA levels for samples stored at 37°C started to decrease after approximately 6 days of storage that corresponded with an increase in particle formation (1–50 μm). Based on maximum free fatty acid release observed at the 48 h time-point, the rituximab formulation showed a significant increase in FFA concentration in the presence of esterase at all storage conditions (Fig. 2D); however, incubation of the rhG-CSF formulation with esterase showed no statistically significant changes in FFA concentration at all storage conditions (Fig. 2C). The observed differences in FFA concentration between two therapeutic protein formulations could be attributed to the differences in polysorbate 80 concentrations in rhG-CSF (0.004% w/v) and rituximab (0.07% w/v) formulations, and the effect of pH (pH 4.0 for rhG-CSF vs pH 6.5 for rituximab formulation) on the enzymatic hydrolysis of polysorbate 80. The higher level of particles in the esterase treated formulations over the untreated ones was sustained over the course of the study. Interestingly, samples treated with an esterase and stored at 4°C showed a similar increase in FFA levels to samples treated in the same way but stored at 37^o^C, but the FFA levels did not decrease at any timepoint up to 6 months of storage. Particle levels remained low in samples stored at 4°C that were either untreated or treated with an esterase.

Commercial Polysorbate 80 NF excipients can exhibit significant variation in fatty acid composition, but the impact of fatty acid as a variable in terms of polysorbate’s role in causing particle formation is not completely understood. Recently, several groups have evaluated the functionality of common and novel polysorbate 80 excipients when subjected to varying mechanical and oxidative stresses (27–29). Kranz et al. evaluated enzyme-mediated hydrolysis of different grades of polysorbate 80 using an esterase from *Saccharomyces cerevisiae*, and found that both the commercial excipient grade of Polysorbate 80, commonly used in US, Europe and Japan, and a novel polysorbate 80 grade synthesized from pure oleic acid were similarly susceptible to enzymatic hydrolysis. However, the novel polysorbate 80 was found to have greater predisposition to oxidative degradation compared to the polysorbate 80 excipient commonly used in the US, Europe and Japan. Another recent study by Grabarek et al. compared different grades of polysorbate 80 and found similar levels of performance when subjected to mechanical stress, such as shaking, free-fall, or syringe pump test (28). A recent study conducted to monitor hydrolytic degradation of polysorbates and formation of particles found homogenous all-oleate PS80 to be less prone to FFA particle formation compared to heterogenous PS80 (30). In alignment with these previous studies, we found that treatment of both types of polysorbate 80 used in this study with an esterase led to rapid hydrolysis as indicated by similar increases in FFA consistent with the composition of fatty acids in the two types of polysorbate we studied, and PS-80 HOA showed less susceptibility to particle formation compared to PS-80 NF (Fig. 3 E and 3G). While we did not evaluate the contribution of mechanical stress or oxidation to degradation of the different grades of polysorbate, we further investigated the propensity for particle formation for each grade of polysorbate.

The treatment with esterase led to greater formulation instability in terms of polysorbate degradation and particle formation, but degradation of polysorbate had no impact on therapeutic protein stability under our experimental conditions tested. Aggregation or degradation of therapeutic proteins may lead to reduction of main band in SDS-PAGE or reduction of main peak in SEC-UPLC; however, no significant changes in protein main bands in the SDS-PAGE (Fig. 4D, 5B) or main peak in SEC-UPLC (4A, 5A) after exposure to enzyme-induced stress conditions for polysorbate degradation confirms that no aggregation or degradation of proteins occurred under our experimental conditions. The two types of polysorbate 80 we studied did not appear to show appreciable differences in terms of rhG-CSF or Rituximab protein instability, even though esterase was capable of hydrolyzing polysorbate 80 to release FFA. Particle formation in polysorbate 80-containing therapeutic formulations increased when stressed by addition of esterase and storage at 37°C (13, 16). Formulations containing PS-80 NF treated with esterase showed greater sub-visible particle formation when compared to formulations containing PS-80 HOA. The critical micelle concentration (CMC) and solubility of FFA depends on the number of carbons in the fatty acid chain and degree of saturation of hydrocarbon bond (31). Therefore, longer chain saturated fatty acids assemble into aggregates at a lower FFA concentration than shorter chain or unsaturated fatty acids (32) (33). The PS-80 NF evaluated in this study is composed of mixture of saturated (C14, C16, C18) and unsaturated fatty acids (C16:1, C18:1, C18:2, C18:3) whereas PS-80 HOA contains the monounsaturated fatty acid (C18:1) at ≥98%), and minimal amounts of the typical saturated fatty acids found in the commercial grade. Therefore, greater number of particles observed in PS-80 NF compared to PS-80 HOA with esterase treatment can be attributed to the release of saturated FFAs from PS-80 NF in the solution (31).

Degradation of the polysorbate can compromise its ability to stabilize biopharmaceuticals and could result in aggregation resulting in loss of therapeutic efficacy or potentially immunogenicity. We used several orthogonal analytical methods to assess protein stability after reformulation with either PS-80 NF or PS-80 HOA. No significant differences in protein aggregations were seen in either formulation. Additionally, biological activity assays did not reveal any difference between formulations prepared with either PS-80 NF or PS-80 HOA. Although these results demonstrate the importance of polysorbate in maintaining the stability of therapeutic proteins as seen by large increases in particle formation, the use of PS-80 HOA and PS-80 NF did not appear to show appreciable differences in terms of rhG-CSF or rituximab protein purity, stability or biological activity. There are several possible explanations for the lack of difference in biological activity and immunogenicity between samples in which polysorbate 80 has undergone hydrolysis and those in which the excipient remained intact, i.e., where there was little or no evidence that hydrolysis has taken place. We used 1 U/mL esterase based on previous publication, but we did not perform additional experiments to demonstrate if increasing esterase concentration will release additional FFA from PS-80 because of complete hydrolysis of fatty acid ester bonds in PS-80. It is possible that in formulations that had undergone some fatty acid hydrolysis sufficient PS-80 was still present to effectively protect the therapeutic protein from any interfacial stress (6). Indeed, previous studies have demonstrated that a 10% loss of polysorbate did not reduce functionality of either compendial or higher purity grades of polysorbate (28). It has also been shown that other physical stressors, such as dropping or even shipping, can lead to particle formation and changes in the therapeutic protein (27, 28, 34, 35). Although our formulations were subjected to elevated temperatures, we did not test the ability of formulations with polysorbate that had undergone hydrolysis to protect the therapeutic protein from additional mechanical stresses. We also did not find significant levels of aggregation or fragmentation for either rhG-CSF (Fig. 4) or rituximab (Fig. 5). It has been suggested that changes to the conformation of the therapeutic protein (aggregation or fragmentation) could be responsible for decreases in therapeutic activity or increases in immunogenicity (36). The lack of significant changes to the therapeutic protein structure observed in this study is one possible explanation for the lack of observed significant difference in biological activity or significant differences in potential immunogenic response. As discussed in result section, although the difference was not significant, a modest trend toward increased IL-1β and CCL2 mRNA induction may suggest a modest risk of inducing activation of PBMCs in some subjects under esterase-induced stress conditions and further studies are warranted.

## Conclusion

Our results obtained under stress conditions suggest that the purity of fatty acid composition in polysorbate 80 may be an important factor in preventing formation of sub-visible particles. Previous reports suggest that particle formation in therapeutic proteins could be a potential quality and safety issue.

The Polysorbate 80 NF used in this study has a mixed fatty acid composition in which oleic acid is the main component as specified by the NF monograph (≥58% oleic acid). We chose to compare a commercial Polysorbate 80 NF excipient with a version prepared by esterification with purified oleic acid (>98%). The formation of greater number of subvisible particles in the formulation containing the commercial excipient (PS-80 NF) may be attributed to the heterogeneity of saturated and unsaturated fatty acid composition compared to the relatively pure monounsaturated oleic acid in PS-80 HOA. Noting that we did not evaluate the fatty acid composition of the hydrolysate or the resulting insoluble fatty acid particles, additional studies would be beneficial to contribute to better understanding of how purity of the fatty acid can be optimized to prevent particle formation in polysorbate-containing formulations. Overall, we conclude that the residual host cell proteases that may be co-purified with therapeutic proteins can trigger the hydrolysis of polysorbate 80 releasing FFA into the formulation, resulting in formation of subvisible particles that contain fatty acid. Otherwise, no obvious detrimental effects on therapeutic protein quality were found in this study. Insoluble particle formation may be sensitive to the degree of saturation in the fatty acids liberated via hydrolysis and their relative solubility in water. This study was limited to investigation of one type of polysorbate, i.e., polysorbate 80. Thus, we did not compare other polysorbate types in which the fatty acids are predominately saturated. The fact that the fatty acid moiety in polysorbate 80 consists of predominately unsaturated fatty acid could explain why we did not see a significant difference in particle formation in the two types of polysorbate 80.

### Acknowledgments and Disclosures

We are thankful to Croda Inc. for providing polysorbate 80 HOA. KS is currently employed by AstraZeneca. This work was funded in part by an appointment to the Research Participation Program at the Office of Biotechnology Products, Office of Pharmaceutical Quality, Center for Drug Evaluation and Research at the U.S. Food and Drug Administration administered by the Oak Ridge Institute for Science and Education through an interagency agreement between the U.S. Department of Energy and the FDA. The views expressed in this article are those of the authors and do not necessarily reflect the official policy or position of the U.S. Food and Drug Administration and the Department of Health and Human Services, nor does mention of trade names, commercial products, or organizations imply endorsement by the U.S. Government. The authors declare that they have no conflicts of interest.

## Abbreviations

ADCC: antibody dependent cellular cytotoxicity
API: active pharmaceutical ingredient
FFA: free fatty acid
HOA: High oleic acid
mAb: monoclonal antibody
MFI: microflow imaging
rhG-CSF: recombinant human granulocyte-colony stimulating factor
PS: polysorbate
pSTAT3: phosphorylated signal transducer and activator of transcription 3
RP-UPLC: reverse phase ultra-performance liquid chromatography
SEC: size exclusion chromatography
SDS-PAGE: sodium dodecyl sulfate-polyacrylamide gel electrophoresis
STAT3: signal transducer and activator of transcription 3
